# Interrogation of the cell wall integrity pathway in *Aspergillus niger* identifies a putative negative regulator of transcription involved in chitin deposition

**DOI:** 10.1016/j.gene.2020.100028

**Published:** 2020-01-28

**Authors:** Tim M. van Leeuwe, Mark Arentshorst, Peter J. Punt, Arthur F.J. Ram

**Affiliations:** a Leiden University, Institute of Biology Leiden, Molecular Microbiology and Biotechnology, Sylviusweg 72, 2333 BE Leiden, the Netherlands; b Dutch DNA Biotech, Hugo R Kruytgebouw 4-Noord, Padualaan 8, 3584 CH Utrecht, the Netherlands

**Keywords:** CWS, cell wall stress, CWI, cell wall integrity, CFW, CalcoFluor White, sgRNA, single guide RNA, PAM, protospacer adjacent motif, DSB, double strand break, SNP, single nucleotide polymorphism, indel, insertion or deletion, GFP, green fluorescent protein, *kusA*, ku70 orthologue *Aspergillus niger*, *pyrG*, Orotidine-5′-phosphate decarboxylase (uracil synthesis), *nicB*, nicotinate mononucleotide pyrophosphorylase (nicotinamide synthesis), *hygB*, Hygromycin B resistance cassette, PHD, plant homeo domain, TFIIS, transcription elongation factor S-II, SPOC, spen paralogue and orthologue c-terminal, Spen, split ends, RNAPII, RNA polymerase II, DR, direct repeat, 5′-FOA, 5-fluoorotic acid, Chitin, Parasexual cross, CRISPR/Cas9, Transcriptional repressor, Cell wall integrity pathway, *BYE1* orthologue

## Abstract

Post-fermentation fungal biomass waste provides a viable source for chitin. Cell wall chitin of filamentous fungi, and in particular its de-*N-*acetylated derivative chitosan, has a wide range of commercial applications. Although the cell wall of filamentous fungi comprises 10–30% chitin, these yields are too low for cost-effective production. Therefore, we aimed to identify the genes involved in increased chitin deposition by screening a collection of UV-derived cell wall mutants in *Aspergillus niger*. This screen revealed a mutant strain (RD15.4#55) that showed a 30–40% increase in cell wall chitin compared to the wild type. In addition to the cell wall chitin phenotype, this strain also exhibited sensitivity to SDS and produces an unknown yellow pigment. Genome sequencing combined with classical genetic linkage analysis identified two mutated genes on chromosome VII that were linked with the mutant phenotype. Single gene knockouts and subsequent complementation analysis revealed that an 8 bp deletion in NRRL3_09595 is solely responsible for the associated phenotypes of RD15.4#55. The mutated gene, which was named *cwcA* (*cell wall chitin A*), encodes an orthologue of *Saccharomyces cerevisiae* Bypass of *ESS1* (*BYE1*), a negative regulator of transcription elongation. We propose that this conserved fungal protein is involved in preventing cell wall integrity signaling under non-inducing conditions, where loss of function results in constitutive activation of the cell wall stress response pathway, and consequently leads to increased chitin content in the mutant cell wall.

## Highlights

• An *Aspergillus niger* UV-mutant with increased cell wall chitin was characterized.
• Causative mutation was identified in a single gene, named cell wall chitin A (*cwcA*).
• CwcA is orthologous to yeast Bye1p and exists as a single copy gene.
• Three relevant domains are found in both CwcA and Bye1p: PHD, TFIIS and SPOC.
• CwcA acts as negative regulator of CWI signaling.

## 1 Introduction

*Aspergillus niger* is a filamentous fungus widely used in industrial fermentations to produce organic acids, enzymes and pharmaceuticals (Meyer et al., 2011; Pel et al., 2007; Punt et al., 2002; Wösten et al., 2013). Specifically, *A. niger* is renowned for its citric acid yields and is able to produce up to 95 kg of citric acid per 100 kg of carbon source, contributing the majority of the worldwide estimate annual yield: 9,000,000 metric tons (Karaffa and Kubicek, 2003). Large scale fermentations result, in addition to the desired product, in accumulation of fungal biomass; a product that is either incinerated or used as a low cost fertilizer for agriculture (Ghormade et al., 2017). However, post-fermentation fungal cell wall biomass waste contains many different sugar polymers that could provide an added-value product. Due to the high levels of post-mycelial biomass produced annually, *A. niger* is considered a fungus of interest to be used of post-fermentation harvesting for cell wall products, such the important biopolymer chitosan with its broad range of applications in different fields (Dhillon et al., 2012).

The fungal cell wall consists of α-glucans (α-1,3-glucans, mixed α-1,3/1-4-glucan and α-1,6-glucans), β-glucans (β-1,3-glucans, β1,6-glucans, mixed β-1,3/1,4 and β-1,3/1,6 varieties), chitin (β-1,4-linked *N*-acetyl-2-amino-2-deoxy-d-glucose), chitosan (β-1,4-linked 2-amino-2-deoxy-d-glucose), galactomannan and glycoproteins (Gow et al., 2017; Ruiz-Herrera and Ortiz-Castellanos, 2019). All fungal cell walls most commonly contain β-1,3-glucans that forms a backbone structure to which other β-glucans, galactomannans or chitin can be cross-linked. Total polymer content and relative composition differs among species and, in addition, is dependent on environmental cues such as nutrients, cultivation conditions, mycelial age, stress or hypoxia (Free, 2013; Lord and Vyas, 2019; Pochanavanich and Suntornsuk, 2002).

Among all cell wall components, chitin and its de-*N-*acetylated derivative chitosan are especially of industrial interest. Chitosan has been reported to have many applications across fields of medicine, cosmetics, agriculture and food industry (Ribeiro et al., 2009; Shen et al., 2009; Takai et al., 2001; Zou et al., 2016). Varying degrees of de-acetylation (DD) and degrees of polymerization (DP) of chitosan regulate its active properties and determine its wide range of applications (El Gueddari et al., 2014). Naturally occurring, cell wall chitosan is most often found among pathogenic species, and is required for both virulence and as a means of avoiding recognition of the immune system in the opportunistic human pathogen *Cryptococcus neoformans* (Baker et al., 2011; Bose et al., 2003). Similarly, phytopathogenic fungi convert cell wall chitin to chitosan that is required for infection. This results in evasion of the plant-host defense response, while simultaneously reducing susceptibility to plant-produced chitinases (Geoghegan et al., 2017). In *A. niger*, chitin and chitosan content have been shown to be dependent on strain, mycelial age, cultivation medium, conditions and extraction methods (Kumaresapillai et al., 2011; Pochanavanich and Suntornsuk, 2002; White et al., 1979). Chitin content has been reported in the range from 10% up to 42% (Knorr, 1991) of the cell wall dry weight, whereas chitosan yields are reported between 5 and 11%, with DD ranging from 73 to 90% (Dhillon et al., 2013; Muñoz et al., 2015; Pochanavanich and Suntornsuk, 2002).

Given the interesting properties of chitin and chitosan, the use filamentous fungi for chitin and chitosan production has been considered. Obviously to make this a profitable option, high levels of cell wall chitin and optimization of chitin extraction are required (Cai et al., 2006a; Dhillon et al., 2012). One approach is to improve the overall chitin content in fungal cell walls. As such, efforts have been made to optimize the production of chitin and its de-*N-*acetylated derivative chitosan through genetic modification of the chitin biosynthetic pathway or through alterations of fermentation conditions (Deng et al., 2005; Hammer and Carr, 2006; Ja'afaru, 2013; Nwe and Stevens, 2004). Alternatively, increased cell wall chitin deposition has been reported to coincide with cell wall stress (CWS) in filamentous fungi (Fortwendel et al., 2010; Guest et al., 2004; Ram et al., 2004). Cell wall stress induced signaling of the cell wall integrity (CWI) pathway in *A. niger* is known to induce expression of genes involved in alpha-glucan and chitin synthesis, *agsA* (alpha-glucan synthase A) (Damveld et al., 2005) and *gfaA* (glutamine-fructose-6-phosphate-amidotransferase A), respectively (Ram et al., 2004). Consequently, CWS could be used to increase the extractable yield of chitin.

We previously reported about a set of cell wall mutants that showed constitutive high levels of *agsA* expression: strains were equipped with a dual reporter system where both an *amdS* and a Histone 2B-GFP (H2B-GFP) construct were fused to the *agsA* promoter (*PagsA*). UV-mutagenesis followed by selection for improved growth on acetamide as a sole nitrogen source, containing H2B-GFP labeled nuclei (selection against *cis*-mutations), allowed to obtain cell wall mutants with a constitutively activated CWI pathway (Damveld et al., 2008). In the study reported here, we specifically screened for cell wall mutants from this collection for increased chitin deposition. Consequently, UV mutant RD15.4#55 was identified that showed constitutive expression of *agsA* and a 30–40% increase in cell wall chitin content. Additional phenotypes of this strain are sensitivity to SDS, also suggesting an effect on cell wall or cell membrane, and the production of an unknown yellow compound. Genome sequencing combined with a classical genetics approach identified mutations in two genes that could be responsible for the mutant phenotypes. Single gene knockouts and complementation studies were used to show that the disruption of NRRL3_09595 (An11g06750), an orthologue of *BYE1* encoding a negative regulator of transcription elongation in *Saccharomyces cerevisiae,* causes an increase in cell wall chitin deposition.

## 2 Materials and methods

### 2.1 Strains, media, growth conditions

Strains used in this study can be found in Table 1. MA169.4 (*cspA1*, *ΔkusA::DR-amdS-DR, pyrG*)*-* (Carvalho et al., 2010) was used for all single knockout transformations. All media were prepared as described by Arentshorst et al., 2012. In all cases (unless otherwise specified) minimal medium (MM) contained 1% (w/v) glucose, 1.5% agar and was supplemented with uridine (10 mM), when required. Complete medium (CM) contained 1% (w/v) glucose, 1.5% agar (Scharlau, Barcelona, Spain), 0.1% (w/v) casamino acids and 0.5% (w/v) yeast extract in addition to MM. To harvest spores, strains were first inoculated from −80 °C glycerol stocks onto fresh CM plates and were allowed to grow and sporulate for 5–7 days at 30 °C. Spores were harvested by addition of 15 mL of 0.9% (w/v) NaCl to CM spore plates and were gently scraped from the plate surface with a cotton stick. Spore solution was pipetted through sterile cotton filters (Amplitude™ Ecocloth™ Wipes, Contec Inc., Spartanburg, SC, USA) to eliminate large mycelial debris. Spore solutions were counted using Bio-Rad TC20™ Automated Cell Counter (Bio-Rad Laboratories, Inc. USA) using Counting Slides, Dual Chamber for Cell Counter (Cat#145–0011, Bio-Rad Laboratories, Inc. USA).

**Table 1:** All strains used in this study.

Name	Genotype	Reference
N402	*cspA1, amdS-*	Bos et al., 1988
MA169.4	*cspA1, ΔkusA::DR-amdS-DR, pyrG-*	Carvalho et al., 2010
RD15.4	cspA1, pyrG-, PagsA-H2B-GFP-TtrpC-pyrG*, *PagsA-amdS-TamdS + pAN7-1* (*hph+*)	Damveld et al., 2008
RD15.8	cspA1, pyrG-, PagsA-H2B-GFP-TtrpC-pyrG*, *PagsA-amdS-TamdS + pAN7-1* (*hph+*)	Damveld et al., 2008
RD15.4#55	UV-mutant RD15.4	Damveld et al., 2008
RD15.8#16	UV-mutant RD15.8	Damveld et al., 2008
RD15.8#35	UV-mutant RD15.8	Damveld et al., 2008
RD15.8#36	UV-mutant RD15.8	Damveld et al., 2008
RD6.13#6	UV-mutant RD6.13	Damveld et al., 2008
RD6.13#7	UV-mutant RD6.13	Damveld et al., 2008
RD6.13#8	UV-mutant RD6.13	Damveld et al., 2008
RD6.13#16	UV-mutant RD6.13	Damveld et al., 2008
RD6.47#56	UV-mutant RD6.47	Damveld et al., 2008
TLF55	RD15.4UV#55, *pyrG-* (5'-FOA selected)	This study
TLF51	RD15.4UV#55, *pyrG-* (5'-FOA selected)*, ΔbrnA*	This study
JN6.2	*cspA1, nicB::hygB, olvA::AOpyrG*	Niu et al., 2016
TLF91	Diploid strain: JN6.2xTLF51(3)	This study
MA841.1	*cspA1, ΔkusA::DR-amdS-DR, pyrG-,* ΔAn04g04020*::AOpyrG*	This study
MA842.1	*cspA1, ΔkusA::DR-amdS-DR, pyrG-,* ΔNRRL3_03052*::AOpyrG*	This study
MA843.1	*cspA1, ΔkusA::DR-amdS-DR, pyrG-,* ΔNRRL3_09002*::AOpyrG*	This study
MA844.1	*cspA1, ΔkusA::DR-amdS-DR, pyrG-,* ΔNRRL3_09595*::AOpyrG*	This study
TLF83	RD15.4UV#55, *pyrG-* (5'-FOA selected), restored NRRL3_09595	This study

### 2.2 Calcofluor White staining and confocal laser scanning microscopy

Strains for microscopy were cultured as described above. Spore solutions were diluted and 10^4^ spores were spotted on plates containing 20 mL MM 1% agarose, and were incubated for 8 h at 30 °C to allow germination. Agar cubes of approximately 1 cm^2^ were excised containing the spot of germlings, and were inverted on top a 24 × 60 mm cover slide containing a 20 μL droplet of 5 μg/mL CalcoFluor White (CFW). Following 5 min of incubation, samples were imaged for CFW fluorescence with a 405 nm laser in a Zeiss Observer confocal laser-scanning microscope (Zeiss, Jena, Germany). Images were processed and analyzed using FIJI (ImageJ) software (Schindelin et al., 2012). To all images, background subtraction was applied (Rolling ball radius 50.0 pixels) prior to processing into *Z*-project with Max Intensity settings. Look-up table used was Cyan Hot.

### 2.3 SDS sensitivity assays

Cell wall disturbing compound SDS was added to MM agar plates from a 10% stock to obtain final concentrations of either 0.004%, 0.0045% or 0.005% SDS. Spores were counted, serially diluted into 2000, 200, 20 and 2 spores/μL and 5 μL of respective dilutions were spotted on MM SDS plates. Plates were incubated for 3–5 days at 30 °C prior to scoring.

### 2.4 Cell wall isolation and chitin analysis

Cell wall isolation, hydrolysis and chitin content analysis, measured as total glucosamine, have been performed as described previously (van Leeuwe et al., 2020). Cell wall glucosamine measurements from independent replicate experiments are expressed as means ± SEM. The statistical analysis was carried out using software R studio (Version 1.1.456) (RStudio: Integrated Development for R. RStudio, Inc., Boston, 2016). For total cell wall glucosamine experiments, we used one-way ANOVA. When there was significant difference between groups, we ran a posthoc Tukey multiple-comparisons analysis. Significance is indicated as *p* > 0.05, not significant (n.s.) *p* ≤ 0.05 (*), *p* ≤ 0.005 (**), *p* ≤ 0.001 (***) and *p* ≤ 0.0001 (****).

### 2.5 DNA isolation, Illumina sequencing and SNP analysis

Genomic DNA was isolated as described by Arentshorst et al., 2012. In case of genome sequencing, this procedure was followed by column purification using the Nucleospin Plant II kit (Machery-Nagel), according to the manufacturer's instructions. Genome sequencing was executed by GenomeScan B.·V (Leiden, The Netherlands). The NEBNext® Ultra DNA Library Prep kit for Illumina (cat# NEB #E7370S/L) was used to process the samples. Fragmentation of the DNA using the Biorupor Pico (Diagenode), ligation of sequencing adapters, and PCR amplification of the resulting product was performed according to the procedure described in the NEBNext Ultra DNA Library Prep kit for Illumina Instruction Manual. The quality and yield after sample preparation was measured with the Fragment Analyzer. The size of the resulting product was consistent with the expected size of approximately 500–700 bp. Clustering and DNA sequencing using the Illumina cBot and HiSeq 4000 was performed according to manufacturer's protocols. A concentration of 3.0 nM of DNA was used. HiSeq control software HCS v3.4.0 was used. Image analysis, base calling, and quality check was performed with the Illumina data analysis pipeline RTA v2.7.7 and Bcl2fastq v2.20. SNP calling was performed according to GenomeScan Guidelines Small Variant Analysis v3.0. The Variant Call Format (VCF) files were manually analyzed by the authors. Frequency score of identical SNP call boundary was set to ≥0.75, while sequencing depth was left unselected.

### 2.6 Parasexual cycle and segregant analysis

Formation of heterokaryons and selection for diploids was performed as described previously (Arentshorst and Ram, 2018). To obtain an auxotrophic haploid derivative of RD15.4#55, this strain was subjected to 5′-FOA counter selection to lose the *pyrG* marker (Arentshorst et al., 2015), resulting in strain TLF55 (Table 1). TLF55 was subsequently than transformed with pFC330_*brnA*-sgRNA (pTLL37.1) and a knockout repair DNA fragment as described previously (van Leeuwe et al., 2019).

The RD15.4#55, *pyrG-, ΔbrnA* strain, TLF51 (Table 1), was cured of the pTLL37.1 plasmid to ensure TLF51 was *pyrG-* for a parasexual cross. Wild type derivative JN6.2 (Table 1) was used as second haploid auxotrophic strain for the parasexual cross. For the parasexual cross, these two haploid strains are coerced to fuse without supplementation for their respective auxotrophic deficiencies. This process yields a heterokaryotic, prototrophic mycelium in which karyogamy can occur at a very low frequency, resulting in a diploid strain. Due to the primarily uninuclear nature of *A. niger* asexual spores, color markers help identify whether nuclei have fused, and become black as a result of complementing alleles from the other chromosome, or remain unfused as one of the individual colors in the heterokaryotic mycelium. An obtained diploid contains both chromosome-sets and can be haploidized to allow random distribution of each chromosome by exposure to benomyl, creating auxotrophic, brown- or olive-colored segregants. Segregation of diploid TLF91 was performed at 0.4 μg/mL benomyl on complete medium (CM) supplemented with 10 mM uridine and 2.5 μg/mL nicotinamide, haplodizing into brown and olive colored segregants. Segregants were single streaked twice on MM with uridine and nicotinamide prior to phenotypic characterization of segregants. Segregation analysis of the cell wall mutant phenotype and auxotrophic markers was performed on MM, MM + uridine and MM + uridine + nicotinamide + 0.005% SDS.

### 2.7 Construction of single gene deletions

MA169.4 (Table 1) was transformed after protoplastation as described previously (Arentshorst et al., 2012) to remove the entire ORF, generating split marker fragments using the split marker approach for single gene knockouts (Arentshorst et al., 2015) with *Aspergillus oryzae pyrG* (*AOpyrG*) as selection marker. Flanks were generated via PCR using N402 genomic DNA as template and primers as described in Supplementary Table 1. *AOpyrG* fragments were obtained using plasmid pAO4-13 (de Ruiter-Jacobs et al., 1989) as template and primers as described in Supplementary Table 1. Through fusion PCR, split marker fragments were created containing *AOpyrG* as selection marker. For transformation, approximately 2 μg of DNA per flank was added to protoplasts. Transformation plates were incubated on MMS for 6 days at 30 °C. Transformed colonies were single streaked on MM twice for purification and were genotyped using diagnostic PCR (data not shown).

### 2.8 Complementation of RD15.4#55 with wild type NRRL3_09595 allele

Complementation of NRRL3_09595 was employed using CRISPR/Cas9 mediated gene editing with a marker-free repair DNA fragment (van Leeuwe et al., 2019). Primers OTL464 and OTL465 were used in combination with pTE1_rev and pTE1_for, respectively, to obtain a sgRNA construct to target the mutant allele NRRL3_09595 in RD15.4#55. Plasmids pTLL108.1 and pTLL109.2 were used as template DNA for sgRNA flanks (van Leeuwe et al., 2019). Cloning of the sgRNA into pFC330 resulted in pFC330_NRRL3_09595-*mut*-sgRNA. Marker-free repair DNA fragment of 449 bp was obtained with OTL385 and OTL386, using N402 as template DNA. Repair DNA fragment contained 308 bp overlap upstream and 141 bp downstream of the double strand break (DSB). CRISPR/Cas9 plasmid transformations were performed after protoplastation of TLF55: 2 μg of Cas9-sgRNA plasmid with 2 μg of repair DNA fragment was used for transformation. Transformation plates were incubated on MMS for 6 days at 30 °C. Transformed colonies were single streaked on selectable medium to select for the presence of the Cas9-sgRNA plasmid. Next, a single colony was picked and transferred to non-selective MM 10 mM uridine medium, allowing loss of the Cas9-sgRNA plasmid. A third streak of a single colony on both MM and MM 10 mM uridine was performed as a control for loss of plasmid. DNA from plasmid-cured strains was isolated as described by Arentshorst et al., 2012, using mortar and pestle to grind the mycelium in liquid nitrogen. Genotypes were confirmed using diagnostic PCR for the 8 bp deletion in the mutant allele of NRRL3_09595. Diagnostic PCR fragments from RD15.4, RD15.4#55 and TLF55 transformants complemented with the wild type NRRL3_09595 allele were sequenced to check for either absence or presence of the 8 bp deletion in the mutant allele (Macrogen Europe, Amsterdam, The Netherlands).

## 3 Results

### 3.1 RD15.4#55 shows increased cell wall chitin, SDS sensitivity and yellow pigment production

**Fig. 1:**
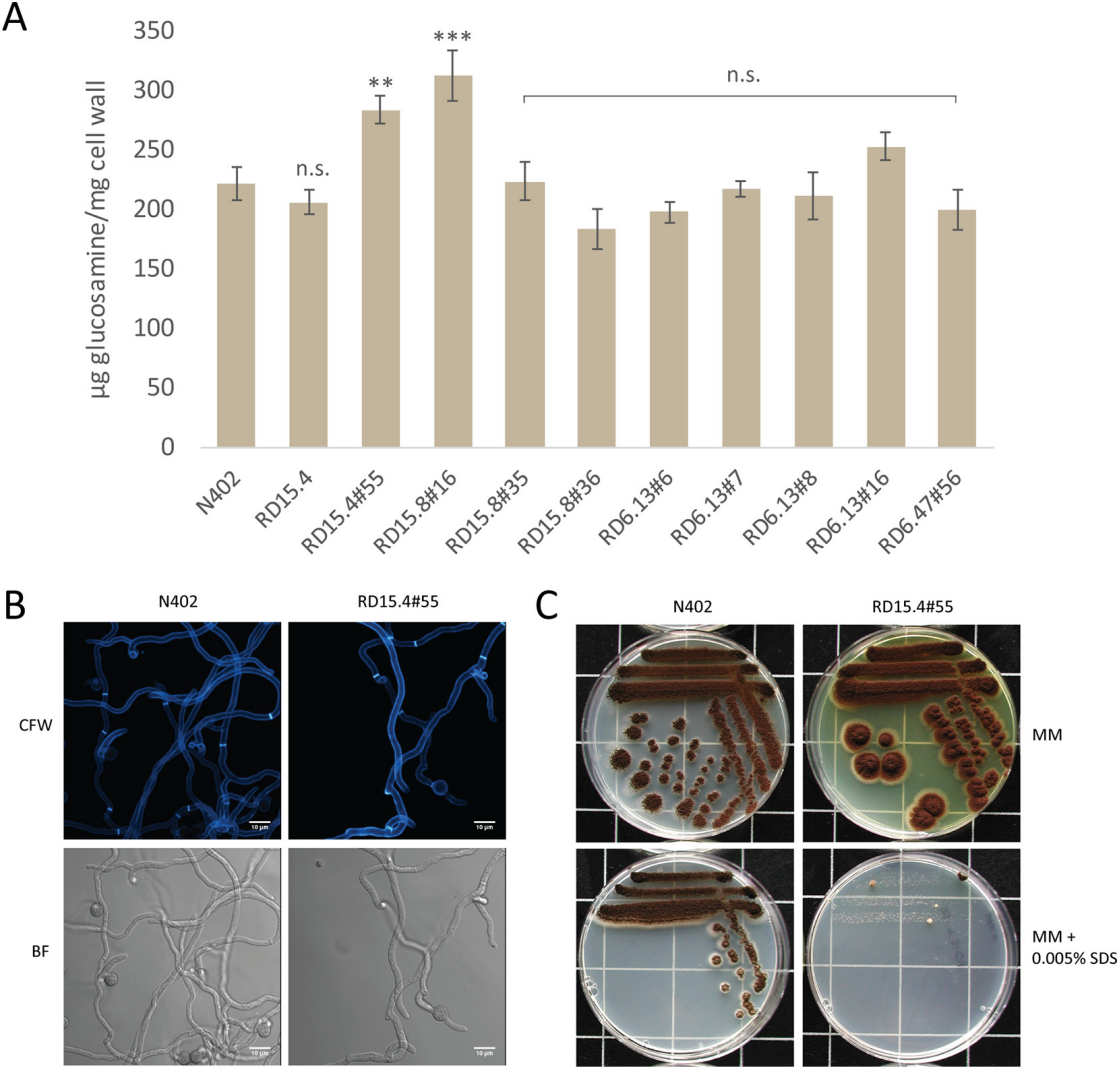
Glucosamine content analysis and candidate selection from UV-mutant collection. (A) Chitin content measured as total glucosamine and normalized to wild type (N402) levels to indicate percentage differences between wild type and listed mutants. Asterisks refer to statistical difference (Section 2.4). (B) CLSM of N402 and RD15.4#55. Strains were grown as described (Section 2.2), top images show chitin staining with Calcofluor White (CFW) and bottom images show bright field (BF) setting. Bars indicate 10 μm (C) Single streak of N402 and RD15.4#55 on MM and MM with 0.005% SDS grown for 96 h at 30 °C. Statistical methods and significance are described in Section 2.4. Listed significant differences are compared to N402.

A previously obtained cell wall stress UV-mutant library (Damveld et al., 2008) was used to screen for mutants with a higher cell wall chitin content, initially using Calcofluor white (CFW) staining followed by chemical quantification of total glucosamine content (see Section 2.4). Nine candidates that exhibited increased CFW staining were analyzed for cell wall glucosamine content. Both RD15.4#55 (283 ± 11.6 μg/mg) and RD15.8#16 (312 ± 20.9 μg/mg) showed a significantly increased cell wall glucosamine content compared to both N402 (221 ± 13.8 μg/mg) and parental strain RD15.4 (205 ± 10.2 μg/mg), displayed in Fig. 1A. Strain RD15.4#55 was selected for further phenotypic assessment, whereas RD15.8#16 is be discussed elsewhere (van Leeuwe et al., 2020). The germination process was analyzed in conjunction Calcofluor White (CFW) staining during the mutant screen. This showed that the hyphal morphology of RD15.4#55 was similar to the parental strain, indicating that its constitutive condition of cell wall stress does not affect growth (Fig. 1B). Although no controlled growth experiments have been conducted to determine growth rates, also under submerged conditions no obvious growth differences were observed. In addition to glucosamine content, we also tested the effect of cell wall disturbing compounds with Calcofluor White (CFW) and SDS (De Groot et al., 2001; de Nobel et al., 2000; Delley and Hall, 1999). Fig. 1C shows single streaks of N402 and RD15.4#55 on MM and MM + 0.005% SDS. As is evident from these sensitivity assays, RD15.4#55 displays sensitivity towards SDS, indicating that either cell wall membrane or cell wall synthesis is perturbed in this mutant. RD15.4#55 did not show clear sensitivity to CFW (data not shown). Lastly, an indicative feature of RD15.4#55 observed on MM plates was the production of a yellow compound into the surrounding agar. Strain RD15.4#55 was selected for genotypic characterization in an attempt to find the underlying mutation(s) causing an increase in cell wall chitin deposition.

### 3.2 Genome sequencing and segregant analysis of RD15.4#55 shows linkage of the phenotype to chromosome VII

To identify the responsible mutation(s) that cause(s) the phenotype of RD15.4#55, we sequenced the genomic DNA of the parental strain RD15.4 and strain TLF51, a *pyrG-, ΔbrnA* derivative of RD15.4#55 (see Section 2.6). Post data processing as described in Section 2.5 revealed a total of 9 SNPs and 31 indels in TLF51 compared to RD15.4. Indels were found in the *pyrG* and *brnA* markers as expected (data not shown). The remaining 9 SNPs and 29 indels are listed in Supplementary Table 2. A total of 5 SNPs and 24 indels were scored to either be intergenic (26) or present in telomeric regions (3). In addition to intergenic SNPs and indels, four SNPs and five indels were found to be inside ORFs and are described in Table 2.

Strain TLF51 was also used to set up a parasexual cross with JN6.2 (*ΔnicB::hygB, ΔolvA::AOpyrG*, Table 1). The diploid strain (TLF91) was haploidized using benomyl to obtain haploid segregants (see Section 2.6). In an initial segregant screen for SDS sensitivity, 23 out of 26 segregants were found to be both SDS sensitive and *nicB+,* suggesting linkage of the SDS sensitivity phenotype to the *nicB* (NRRL3_09250) locus. Analysis of an additional 80 *nicB*+, segregants showed that 79/80 were SDS sensitive, confirming this linkage analysis of the SDS sensitive phenotype of RD15.4#55 to *nicB*, located on chromosome VII. Because of the parasexual cross (i.e. no meiosis), crossover events are rare (mitotic) and chromosomes are generally fully inherited from either wild type or mutant. Linkage to *nicB*+ (TLF51 chromosome VII) therefore suggests involvement of SNPs located on this chromosome. SNP analysis of TLF51 revealed that both *agsC* and NRRL3_09595 were mutated, resulting in premature translation stop, and are located on chromosome VII (Table 1).

**Table 2:** SNPs found inside ORF of TLF51 (derivative of RD15.4#55, Table 1).

Chromosome	NRRL3 ID	CBS513.88 ID	Gene description	Gene orientation	WT SNP	RD15.4#55 SNP	Alteration in gene product
chr_3_1	NRRL3_03052	An12g08790	Phospholipid translocating ATPase (flippase)	−	G	A	Ser^1426/1519^ → Phe^1426/1519^
chr_3_2	NRRL3_03881	An12g04630	Oxalate decarboxylase	+	C	CT	Insertion at the +21 position of intron 2 (129 bp)
chr_5_2	NRRL3_06613	An16g09230	Putative fungal transcription factor	−	G	GA	Insertion at the +32 position of intron 7 (85 bp)
chr_6_1	N/A	An04g04020	Unknown protein	−	T	A	Phe^77/136^ → Ile^77/136^
chr_7_1	NRRL3_09002	An12g02450	α-glucan synthase C (*agsC*)	+	C	T	Gln^1599/2407^ → Stop^1599/2407^
chr_7_2	NRRL3_09595	An11g06750	*BYE1* orthologue (Transcription elongation factor SII)	−	CGCGGAGGA	G	Frameshift → stop^642/943^
chr_8_2	NRRL3_10506	An18g04180	40S ribosomal protein S19	+	G	GA	insertion at the +31 position of intron 3 (78 bp)
chr_8_2	NRRL3_11411	An08g08570	Hypothetical protein (DNA binding prediction)	−	G	A	Synonymous Valine substitution: GTC to GTT

### 3.3 Single gene knockouts show that ΔNRRL3_9595 displays the same phenotype as RD15.4#55

Based on the linkage analysis we opted to construct full gene knockouts of chromosome VII located genes alpha glucan synthase C (*agsC*) and NRRL3_09595, and test how they relate to the phenotypes of RD15.4#55. An additional 3 indels in intergenic regions (Supplementary Table 2) of chromosome VII were left out for consideration. Strain MA169.4 (Table 1) was transformed with split marker flanks, harboring the *AOpyrG* selection marker as described in Section 2.7, resulting in knockout strains MA843.1 (*ΔagsC*) and MA844.1 (ΔNRRL3_09595) (Table 1). Strains were confirmed to have replaced ORFs through diagnostic PCR (data not shown).

**Fig. 2:**
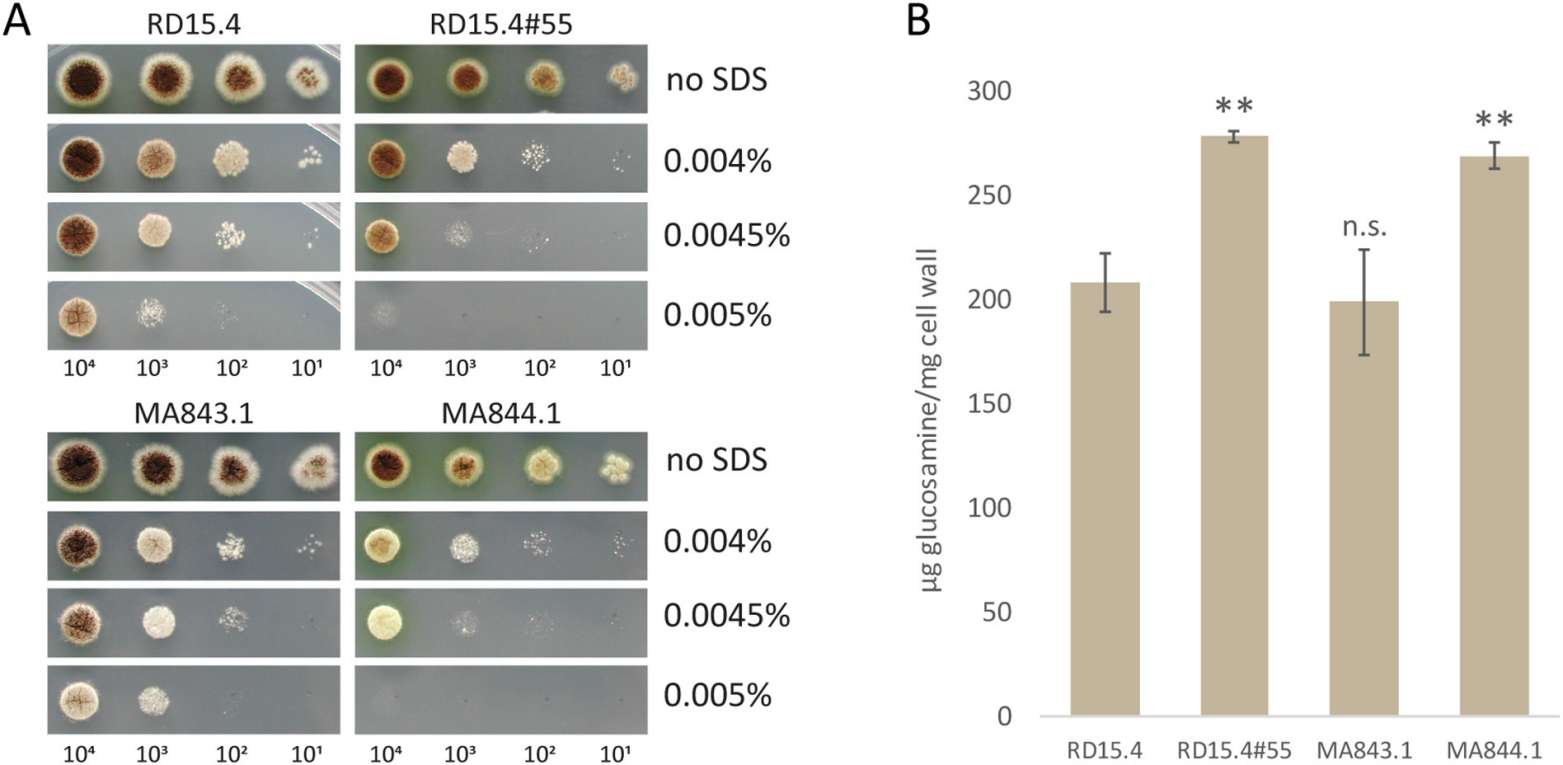
SDS sensitivity and cell wall glucosamine phenotypes of single knockout strains. (A) Growth phenotype and SDS sensitivity of parental strain RD15.4, UV-mutant RD15.4#55 and knockout strains of agsC (MA843.1) and NRRL3_09595 (MA844.1) on MM with 10 mM uridine (U). Strains were grown on either 0.004%, 0.0045% or 0.005% SDS plates. All plates were incubated for 72 h at 30 °C. Spore amounts (#) per spot from left to right are 10^4^, 10^3^, 10^2^ or 10^1^, and are listed below the figure. (B) Total cell wall glucosamine determination of single knockouts. Parental strain RD15.4, UV-mutant RD15.4#55 and knockout strains MA843.1 and MA844.1. Cell wall glucosamine of all strains grown in Complete medium (CM) with 10 mM uridine at 30 °C for 17 h (*n* = 3). Statistical methods and significance are described in Section 2.4. Listed significant differences are compared to RD15.4.

Parental strain RD15.4 and UV-mutant RD15.4#55 were cultured together with knockout strains and were plated on MM + uridine containing either 0.004%, 0.0045 or 0.005% SDS plates. Fig. 2A shows that knockout strain MA843.1 (*ΔagsC*) grows similar to the parental RD15.4 strain on both MM and MM + SDS, whereas the growth of MA844.1 (ΔNRRL3_09595) is affected by the presence of SDS, similar to RD15.4#55.

Next, all strains were cultured for cell wall isolation and determination of total glucosamine content (see Section 2.4). Evidently, parental strain RD15.4 was measured to contain a glucosamine content of 208 ± 5.5 μg/mg cell wall, whereas the UV-mutant RD15.4#55 showed a glucosamine content of 278 ± 4.4 μg/mg cell wall: a 33.6% increase in overall glucosamine (Fig. 2B). Single knockout strains MA843.1 (*ΔagsC*) and MA844.1 (ΔNRRL3_09595) were measured to have a glucosamine content of 199 ± 6.2 and 269 ± 5.2 μg/mg cell wall, respectively. Statistical analysis showed that there is a significant difference between RD15.4 and RD15.4#55 and MA844.1, but not between RD15.4 and MA843.1. Neither do RD15.4#55 and MA844.1 differ significantly from each other, suggesting they produce equal, yet increased, levels of cell wall glucosamine.

### 3.4 Complementation of RD15.4#55 with the wild type NRRL3_09595 allele restores all associated phenotypes

**Fig. 3:**
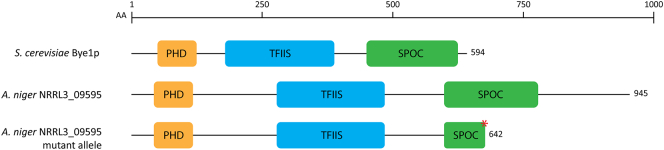
Schematic representation of NRRL3_09595 and respective domains and the yeast orthologue BYE1. Proteins contain a Plant Homeo Domain (PHD, orange), Transcription elongation Factor S-II (TFIIS) superfamily domain (blue) and a Spen paralogue and orthologue C-terminal (SPOC) domain (green). Red asterisk shown for mutant allele of NRRL3_09595 represents an early STOP codon as a result of a frameshift leading to truncation of NRRL3_09595 (see Section 3.4). An amino acid (aa) scale indicates length of proteins. (For interpretation of the references to color in this figure legend, the reader is referred to the web version of this article.)

**Supplementary Fig. 1:**
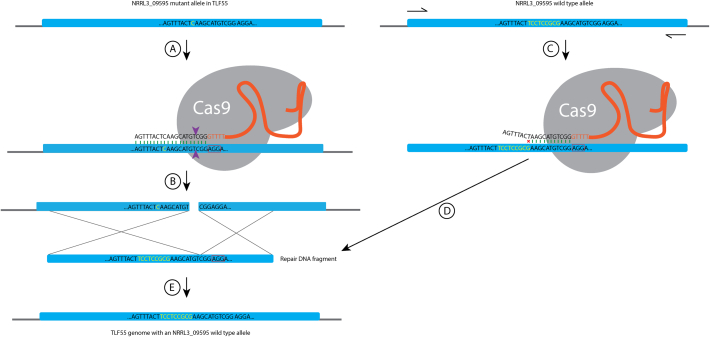
CRISPR/Cas9 design for targeting of the NRRL3_09595 mutant allele for complementation in the TLF55 (RD15.4#55, *pyrG-*) background. (A) NRRL3_09595-*mut*-sgRNA-Cas9 ribonucleoprotein (RNP) complex binds the genomic DNA of the TLF55 strain. Green bars represent matching of bps between the sgRNA target and genomic sequence. PAM site is highlighted by a red box (AGG). Purple arrows indicate the site of the double stranded break (DSB). (B) Creation of a DSB in mutant allele NRRL3_09595. (C) PCR amplification of the NRRL3_09595 wild type allele to generate a repair DNA fragment from that contains 8 bps that are not present in the NRRL3_09595 mutant allele, shown in yellow. As such, the repair DNA fragment is not recognized by the NRRL3_09595-*mut*-sgRNA-Cas9 RNP due to mismatches succeeding from the 12th nucleotide (nt) upstream from the PAM site, displayed by a red cross. (D) The repair DNA fragment from the NRRL3_09595 wild type allele remains intact and allows repair of the NRRL3_09595 mutant allele through homology directed repair. (E) The resultant transformation restore the NRRL3_09595 mutant allele in TLF55 to a wild type allele at the endogenous locus.

The 8 bp deletion found in NRRL3_09595 (*BYE1* orthologue) causes an initial aa change of Ser^636/945^ to Gln^636/945^, followed by a frameshift 5 amino acids (aa) downstream, ultimately leading to stop codon at aa642/945 (Fig. 3). We showed that a full knockout of NRRL3_09595 results in the same phenotype as the RD15.4#55 including SDS sensitivity, cell wall glucosamine and yellow pigment production. To rule out the involvement of other SNPs in RD15.4#55 that may contribute to the phenotype, complementation was performed in the RD15.4#55 strain by introducing the wild type allele of NRRL3_09595 through transformation. CRISPR/Cas9 mediated genome editing was used to introduce the wild type allele at the location of the mutant allele of NRRL3_09595 in RD15.4#55. The 8 bp deletion in the mutant allele of NRRL3_09595 provided a location for the design of a sgRNA target (5′ – AGTTTACTCAAGCATGTCGG – 3′) that is unique for the RD15.4#55 strain. PAM site 5′ – AGG – 3′ is present in both RD15.4 and RD15.4#55, but the target sequence only matches the first PAM-adjacent 11 bp in the RD15.4 (5′ – nnnnnnnnnnAAGCATGTCGG – 3′) due to the deletion. Contrarily, the full target sequence is only found at mutant allele of NRRL3_09595 in RD15.4#55, allowing specific targeting of the mutant allele without recognition of the wild type NRRL3_09595 allele presented as repair DNA fragment (See Supplementary Fig. 1 for a detailed visual representation). The designed target was cloned into a sgRNA expression cassette to obtain pFC330_NRRL3_09595-*mut-*sgRNA (pTLL103.2) as described in Section 2.8, and a 449 repair DNA fragment was amplified from the wild type allele of NRRL3_09595 in RD15.4. A *pyrG-* derivative of RD15.4#55 (TLF55) was transformed with both plasmid pFC330_NRRL3_09595-*mut-*sgRNA and repair DNA fragment.

Single streaks of transformed colonies were checked for the presence of the yellow pigmentation. Yellow pigment production was expected to be absent for transformed strains that successfully incorporated the repair DNA fragment at the NRRL3_09595 locus, whereas the yellow pigment remained visible for transformed strains that did not incorporate the repair DNA fragment. Fifteen transformants were picked from the initial transformation plate, four of which were found to lack yellow color production. Single streaking on MM with uridine removed selection pressure to cure the Cas9 plasmid. This was successful for two out of the four transformants. A diagnostic PCR of the NRRL3_09595 locus, followed by sequencing revealed that the wild type NRRL3_09595 allele had correctly replaced the mutant allele in the TLF83 transformant.

**Fig. 4:**
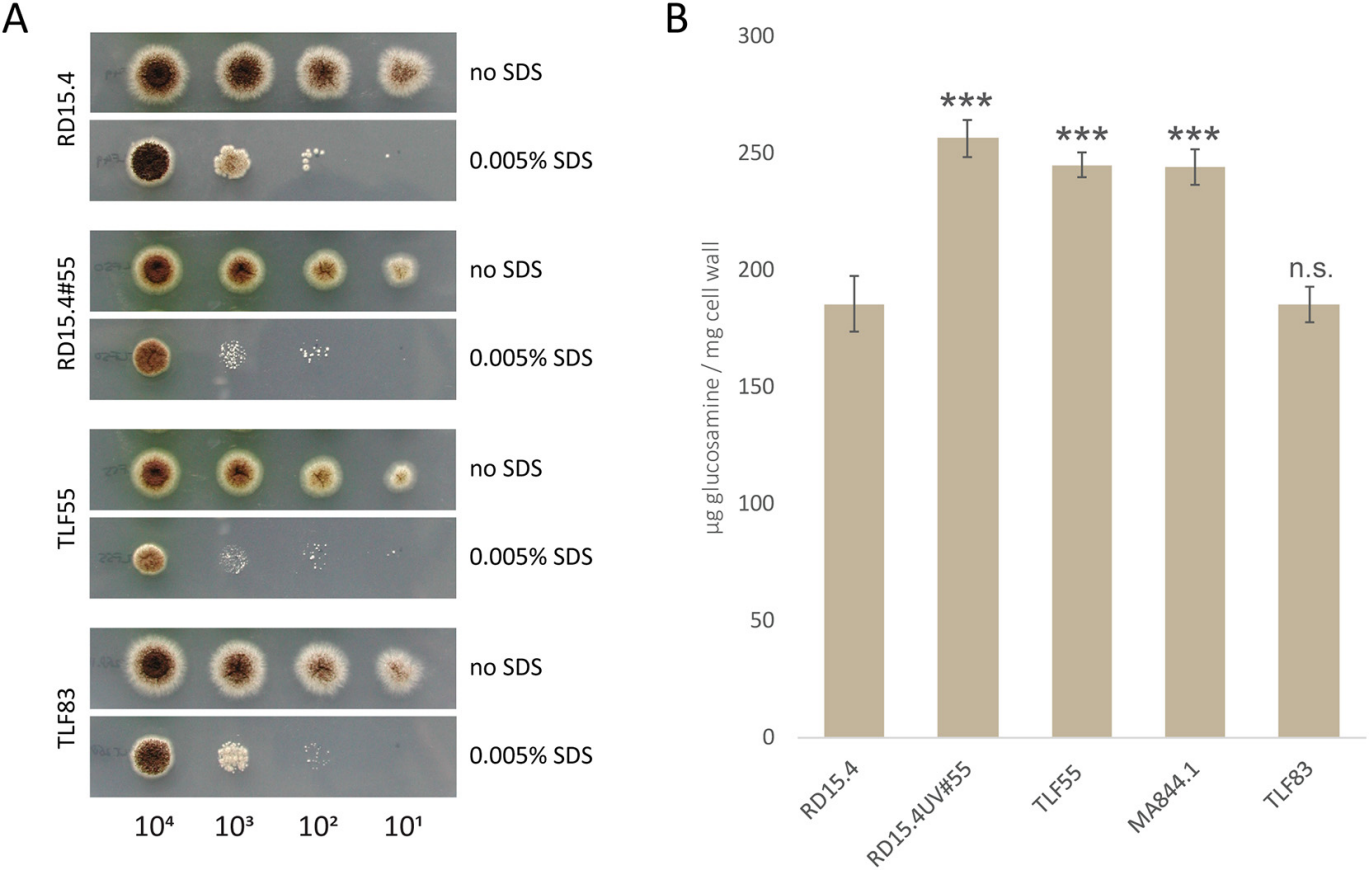
SDS sensitivity and cell wall glucosamine phenotypes. Parental strain RD15.4, UV-mutant RD15.4#55, pyrG- derivative of RD15.4#55 (TLF55), single knockout of NRRL3_09595 (MA844.1) and TLF55 with complemented with wild type NRRL3_09595 (TLF83). (A) Growth on MM with 10 mM uridine and MM with 10 mM uridine and 0.005% SDS. Grown for 72 h at 30 °C. (B) Total cell wall glucosamine strains (*n* = 3). Statistical methods and significance are described in Section 2.4. Listed significant differences are compared to RD15.4.

To show that restoration of NRRL3_09595 in RD15.4#55 (TLF83) restores all associated phenotypes, we cultured RD15.4, RD15.4#55, RD15.4#55, *pyrG-* (TLF55) and TLF83 to create spore solutions of equal age. Spores were diluted and plated on MM with uridine and MM with uridine +0.005% SDS (Fig. 4A) The complemented strain (TLF83) resembles the phenotype of RD15.4, in terms of colony morphology, yellow pigment production and SDS sensitivity. Next, all four strains were cultured together with MA844.1 (ΔNRRL3_09595) to check the total cell wall glucosamine content. Strains were grown for 17 h on CM with uridine as biological triplicates and cell walls were isolated and lyophilized. Fig. 4B shows cell wall glucosamine levels of all tested strains. It is clear from the graph that RD15.4#55, TLF55 and MA844.1 show that there is a significant increase in glucosamine content compared to the parental RD15.4 strain, as previously confirmed. However, all these strains also show a significant difference in cell wall glucosamine compared to TLF83 (restored NRRL3_09595 in RD15.4#55), whereas RD15.4 and TLF83 display identical levels of glucosamine. Taken together with the morphology and SDS sensitivity data, this confirms that NRRL3_09595 is responsible for all the associated phenotypes of RD15.4#55. Based on its effects on levels of cell wall chitin, the NRRL3_09595 gene is named *cwcA* (***c***ell ***w***all ***c***hitin).

## 4 Discussion

In this study, we describe the mutant RD15.4#55 that was selected from a previously obtained forward genetics screen. UV mutagenesis was used in combination with a dual-reporter system, based on *agsA* expression during the CWI response, to screen for mutants that displayed a continuous state of cell wall stress (Damveld et al., 2008). In addition to alpha-glucan, increases of cell wall chitin deposition have been also been reported in fungi through induction of the CWI signal transduction pathway (Fortwendel et al., 2010; Heilmann et al., 2013; Ram et al., 2004; Walker et al., 2015, Walker et al., 2008). In *A. niger*, both *agsA* and *gfaA* have been shown to be induced in response to cell wall stress in an *rlmA* dependent way (Damveld et al., 2005). In the attempt to identify mutants with increased chitin deposition, we screened a set of mutants with increased *agsA* expression for concomitant increases in cell wall chitin. Cell wall analysis showed that RD15.4#55 has increased glucosamine levels compared to wild type strains. Next to an approximate increase in glucosamine content (here, chitin and chitosan) of about 30–40%, RD15.4#55 was found to display sensitivity towards SDS and showed the secretion of an unknown yellow pigment (Fig. 1).

To identify the genotype related to RD15.4#55's phenotypic traits, we carried out a classical genetics approach: RD15.4#55 was prepared for a parasexual cross (Arentshorst and Ram, 2018) with a wild type strain (JN6.2, Table 1) by introducing the *pyrG-* auxotrophic deficiency and disruption of the *brnA* gene as a color identifier (TLF51, Table 1). Segregants from the parasexual cross showed 96.2% linkage of the SDS sensitive phenotype to chromosome VII (*nicB* auxotrophic marker). Chromosome VII harbors both a SNP in NRRL3_09002 (*agsC*) and an 8 bp deletion in NRRL3_09595, now named *cwcA,* that were identified by whole genome sequencing of strain TLF51. Interestingly, SDS sensitivity assays showed that a full deletion of *cwcA* resembled the phenotype of RD15.4#55, whereas a deletion of *agsC* did not. In addition, total cell wall glucosamine analysis revealed that a deletion of *cwcA* causes elevated levels of cell wall glucosamine. The lack of involvement of *agsC* in the cell wall mutant phenotype, although it encodes a potential cell-wall modifying enzyme, is corroborated by the fact that *agsC* shows low expression during vegetative growth, and is not induced by activation of the cell wall integrity pathway (Damveld et al., 2005).

To confirm that only a mutation in *cwcA* was responsible for the chitin disposition phenotype of RD15.4#55, the *cwcA* mutant allele in RD15.4#55 was restored by introducing the wild type allele using CRISPR/Cas9 gene editing at the endogenous genomic location, leaving all other SNPs intact. A SDS sensitivity assay showed that complementation in TLF83 results in wild type sensitivity, and that the SDS phenotype is attributable to *cwcA* (Fig. 4A). Moreover, also cell wall glucosamine analysis clearly showed that *cwcA* is solely responsible for the increase in glucosamine content of RD15.4#55 (Fig. 4B).

Despite a relatively late truncation of the CwcA protein as a result of the frameshift in the mutant allele (Fig. 3), a full knockout resembles the phenotype of RD15.4#55 suggesting that the C-terminal domain is critical for complete function of the protein. DELTA-BLAST analysis revealed that CwcA encodes a 945aa protein that is a putative orthologue of *BYE1* in *S. cerevisiae* (Bypass of ESSential gene 1), containing three functional domains: a Plant Homeo Domain (PHD) finger (aa59-108), a Transcription elongation Factor S-II (TFIIS) superfamily domain (aa269-482) and a Spen (*Split Ends*) Paralogue and Orthologue C-terminal (SPOC) domain (aa617-771). The latter domain is largely absent for the protein product of mutant allele *cwcA,* and may be of importance for the function of this protein. To the best of our knowledge, no literature exists on the function of SPOC domains in filamentous fungi. Spen proteins or SPOC domain containing proteins were first described in *Drosophila melanogaster* and are involved in developmental signaling in embryonic development, where either deletion or mutation of the SPOC domain results in severe perturbation of cell fate specification (Kolodziej et al., 1995; Wiellette et al., 1999). Since then, structural studies have revealed the SPOC domain contains a β-barrel that resembles the *ku80* protein. This domain has been shown to be implicated in protein-protein interactions, also harboring a conserved, relatively basic surface and is suggested to interact with DNA (Ariyoshi and Schwabe, 2003; Lee and Skalnik, 2012). DELTA-BLAST alignment shows that both Pezizomycotina and Saccharomycotina species contain a single orthologue of *cwcA* with all three PHD, TFIIS and SPOC domains, whereas other hits encompassing putative paralogues are evidently deficient in SPOC domains, but still harbor either PHD and TFIIS domains together or either one separately. To date, the function of these single or bi-domain proteins in filamentous fungi is unknown.

In yeast, *BYE1*, the orthologue of CwcA (DELTA-BLAST, 98% query coverage, 16.29% protein identity), has been studied in detail; however no studies have reported on its role in CWS or chitin deposition. Genetic interaction studies have shown Bye1p can act as a multi-copy suppressor on prolyl isomerase *ESS1* (Hanes et al., 1989). *ESS1* is an essential gene required for the phosphorylation of RNA polymerase II (RNAPII) C-terminal domain and affects co-factor binding through conformationally induced changes that may facilitate proper transcription initiation, elongation and termination (Ma et al., 2012). It was shown that Ess1p opposes the positive effects of known elongation factors Dst1p and the Spt4p-Spt5p complex, and it was found that high levels of Bye1p (as multi-copy suppressor) eliminate the requirement of Ess1p, hence Bypass of *ESS1*, suggesting they both act as negative regulators in transcription (Wu et al., 2003). Bye1p interacts with RNAPII through its TFIIS domain and occupies the 5′-region of active genes, and binds posttranslationally modified histone H3 lysine 4 tri-methylation tails (H3K4-3me) of active transcription, using the PHD domain (Kinkelin et al., 2013; Pinskaya et al., 2014). Although *BYE1* is a non-essential gene, it is associated with a very pleiotropic phenotype both when disrupted and when overexpressed (Breslow et al., 2008; Cai et al., 2006b; Kapitzky et al., 2010; Pir et al., 2012; Sopko et al., 2006). Deletion did not cause impaired growth rate (Breslow et al., 2008), similar as we observed for RD15.4#55 (Section 3.1), whereas overexpression in yeast caused slower vegetative growth compared to wild type (Sopko et al., 2006; Yoshikawa et al., 2011). Based on the available data of *BYE1* we propose that CwcA is also involved in DNA binding and has a general function in transcription repression. In this work, we show for the first time that *A. niger BYE1* orthologue, *cwcA*, may act as a transcriptional repressor of the CWI pathway. Additionally, CwcA may be involved in the repression of secondary metabolite clusters as is suggested by the unscheduled production of an unknown yellow compound in the mutant strain.

Previously, the cell wall mutant library as described by Damveld et al., 2008 has provided a rich research tool to discover proteins involved in the CWI pathway of *A. niger*. Genes required for the synthesis of galactofuranose (*ugmA and ugeA*) vacuolar H(+)-ATPase (*vmaD*), and general transcription repressor *tupA* (yeast Tup1 homologue) were all identified from this cell wall mutant library (Damveld et al., 2008; Park et al., 2014; Schachtschabel et al., 2013, Schachtschabel et al., 2012). In case of *ugmA*, it was also found that cell wall chitin was increased by activation of the CWI pathway as consequence of losing cell wall galactofuranose (Afroz et al., 2011; Park et al., 2016). Contrarily, the *tupA* mutant included in the initial cell wall chitin screen presented here (RD15.8#36, Fig. 1A), did not show an increase in cell wall glucosamine. Essentially, *cwcA* and identified genes from these previous studies can be categorized as either cell wall biosynthesis, remodeling/recycling proteins or as regulatory elements. Similar to TupA, CwcA appears to be involved in transcriptional regulation rather than cell wall biosynthesis directly. In addition, both deletion strains display distinct pigment production, indicative of secondary metabolite cluster activation. Despite this generalized comparison, it appears as if these regulatory proteins are most likely involved at different levels of regulation in (repression of) production of multiple cellular processes, including the deposition of cell wall chitin.

## 5 Conclusion

In summary, we show that deletion of CwcA results in a 30–40%increase in total cell wall glucosamine, making a *cwcA* mutant a potential candidate for improved chitin and chitosan production as value added by-product from fungal biomass derived from industrial fungal fermentation.

The following are the supplementary data related to this article.

Supplementary Table 1 All primer used in this study. Supplementary Table 1

Supplementary Table 2 All indels and SNPs found in TLF51 compared to RD15.4. Supplementary Table 2

## Ethics approval and consent to participate

Not applicable.

## Consent for publication

Not applicable.

## Funding

This work is part of the “FunChi” ERA-IB project with project number ERA-IB-15-080, which is (partly) financed by the Dutch Research Council (10.13039/501100003246NWO).

## CRediT authorship contribution statement

**Tim M. van Leeuwe:** Investigation, Formal analysis, Methodology, Writing - original draft, Visualization. **Mark Arentshorst:** Investigation. **Peter J. Punt:** Conceptualization, Writing - review & editing, Funding acquisition. **Arthur F.J. Ram:** Supervision, Conceptualization, Writing - review & editing, Funding acquisition.

## Declaration of competing interest

The authors declare that they have no known competing financial interests or personal relationships that could have appeared to influence the work reported in this paper.
